# Left Atrial Appendage Mechanical Dispersion Assessed by Speckle-Tracking Echocardiography as a Determinant of Left Atrial Appendage Blood Stasis in Patients With Atrial Fibrillation

**DOI:** 10.3389/fcvm.2022.905293

**Published:** 2022-06-06

**Authors:** Yankai Mao, Huajie Zhao, Chan Yu, Yuan Yang, Mingming Ma, Yunhe Wang, Ruhong Jiang, Bowen Zhao, Zhelan Zheng, Chenyang Jiang

**Affiliations:** ^1^Department of Diagnostic Ultrasound & Echocardiography, Sir Run Run Shaw Hospital, Zhejiang University School of Medicine, Hangzhou, China; ^2^Department of Echocardiography and Vascular Ultrasound Center, First Affiliated Hospital, Zhejiang University School of Medicine, Hangzhou, China; ^3^Department of Cardiology, Key Laboratory of Cardiovascular Intervention and Regenerative Medicine of Zhejiang Province, Sir Run Run Shaw Hospital, School of Medicine, Zhejiang University, Hangzhou, China

**Keywords:** left atrial appendage, mechanical dispersion, speckle-tracking echocardiography, atrial fibrillation, left atrium

## Abstract

**Aims:**

We sought to investigate the relationship of left atrial appendage (LAA) mechanical dispersion (MD) with LAA dense spontaneous echo contrast (SEC) or thrombus, and to compare its usefulness in the identification of thrombogenesis with left atrial (LA) MD or LA/LAA strain parameters in patients with atrial fibrillation (AF).

**Methods:**

We conducted a cross-sectional study of 493 consecutive patients with AF [65(58.5–71.0) years, male 66.9%] who underwent echocardiography prior to catheter ablation. We measured the LAA and LA global longitudinal strain (GLS) using speckle-tracking echocardiography (STE). LAA MD and LA MD was defined as the standard deviation (SD) of time to peak positive strain corrected by the R-R interval.

**Results:**

Patients with LAA dense SEC/thrombus (*n* = 70) had significantly higher LAA MD than controls (*n* = 423) [median 14.2(11.6–16.8)% vs 9.4(6.2–12.1)%, *p* < 0.01]. Multivariable analysis showed that LAA MD was independently associated with LAA dense SEC/thrombus in four different models (Odds ratio, 1.23–1.24; *p* < 0.01), and provided additional diagnostic value over clinical and standard echocardiographic parameters. Whereas, LA MD was not independently associated with LAA dense SEC/thrombus and had no incremental value over other LA/LAA mechanical parameters.

**Conclusion:**

LAA mechanical dispersion was an independent determinant of LAA dense SEC/thrombus in AF patients, incremental to conventional risk factors and superior to LA mechanical dispersion.

## Introduction

Atrial fibrillation (AF) is the most common clinically relevant cardiac arrhythmia, posing patients at higher risk of ischemic stroke (1). Importantly, AF-related stroke is associated with an increased mortality and worse outcomes than non-AF strokes (2). Preventive strategies are essential and should be delivered based on risk stratification. CHA2DS2-VASc scoring system is the most prevalent scheme for stratifying stroke risk in AF patient (3), but there is a lack of direct mechanistic link with stroke and its predictive power is modest in those with a score of <2 (4).

For the past century, left atrial appendage (LAA) has been regarded to be the major source of AF-related strokes (5, 6), as more than 95% of thrombus formation originated from LAA (7). Hence, it would be of great clinical significance to identify individuals at high risk of LAA thrombus, especially those with low CHA2DS2-VASc score. The structural and functional remodeling of LAA and LA during AF, including cavity dilation, endocardial fibroelastosis and depressed myocardial function are all potential markers of LAA thrombus and spontaneous echo contrast (SEC) (8–12). Besides the well-established predictors like LA enlargement, LAA morphology and LAA peak flow velocities (13–15), subclinical myocardial dysfunction of LAA and LA detected with speckle-tracking echocardiography (STE) (16) has emerged as useful markers. Impaired LA and LAA global longitudinal strain (GLS) are closely correlated with LAA blood stasis (17–21). STE can also measure the timing of myocardial contraction, known as mechanical dispersion (MD), which represents the degree of discoordination of wall motion. Recently, the role of LA MD in thrombogenesis was investigated as a further step to dissect the mechanism when LA function is depressed, and it was proved to have incremental values in identifying LAA thrombi or sludge (22, 23) and previous stroke/transient ischemic attack (TIA) in AF patients (24, 25). Similarly, we found LAA MD is greater in AF patients with a history of thromboembolism than those without in our previous studies (25, 26), supporting the hypothesis that LAA dyssynchrony may play a central role in the mechanism of thrombogenesis. Whereas, the direct mechanistic link between LAA MD and LAA blood stasis has not been fully addressed. The purpose of this study was to quantify the association of LAA mechanical dispersion with LAA dense SEC or thrombus and to compare its value in the risk stratification for thrombogenesis with LA MD or LA/LAA GLS in patients with AF.

## Materials and Methods

### Study Population

We prospectively included 656 consecutive AF patients without significant valvular diseases or prosthetic valves. All patients were referred for catheter ablation to one of two Chinese tertiary hospitals (Sir Run Run Shaw Hospital, Zhejiang University School of Medicine and First affiliated hospital, Zhejiang University School of Medicine) between April 2019 and May 2021. Exclusion criteria: (1) cardiomyopathy, (2) congenital heart disease, (3) history of any cardiac surgery and/or cardiac device implantation, (4) cardiac mass, (5) inadequate image quality to perform strain analysis, and (6) sinus rhythm at the time of echocardiography. Patients were classified as having either paroxysmal or persistent AF according to the guidelines (3, 27). Clinical information including demographic data, medical history, medication history, and baseline examination data were comprehensively assessed and CHA2DS2-VASc score was computed accordingly (3). Venous blood samples were obtained from the basilic vein after overnight fast. Laboratory data, including homocysteine, lipid levels were collected and analyzed.

The study protocol was approved by the institutional medical ethics committees of the two participating hospitals and was conducted in accordance with the Declaration of Helsinki and its later amendments. All patients provided their written, informed consent.

### Standard Echocardiography

All participants routinely underwent transthoracic echocardiography (TTE) and transesophageal echocardiography (TEE) after admission. Echocardiographic examinations were performed using a Vivid E95 scanner (GE Vingmed Ultrasound AS, Horten, Norway) equipped with a M5Sc (1.4–4.6 MHz) and 6VT (3.0–8.0 MHz) probe. The grayscale frame rate was set to 60–90 frames/second. Standard echocardiographic measurements were taken according to current recommendations (28). LA and LAA volumes (LAV and LAAV) were determined by modified Simpson’s method from apical four- and two-chamber views on TTE (LAV) and two orthogonal views typically at 45° and 135° on TEE (LAAV), respectively. The LA and LAA emptying fraction (LAEF and LAAEF) were calculated as [*maximumvolume*(Vmax)−*minimalvolume*(Vmin)]/Vmax*100%. LAA peak emptying velocity (EV) and filling velocity (FV) was obtained with the sampling placed in the proximal third of the LAA cavity. LAA and the inlet of the pulmonary veins were excluded from LA tracing. All volumetric variables were subsequently indexed by body surface area.

Lidocaine hydrochloride spray was used for local anesthesia before TEE studies. LAA were carefully examined for the presence of dense SEC or thrombus by sweeping from 0° to 180° at the mid-esophageal position. The dense SEC was defined as very slow swirling smoke-like echoes detectable within LAA throughout the cardiac cycle. A thrombus was defined as a fixed or mobile, irregularly shaped, echo-dense mass that was clearly distinct from adjacent endocardium and pectinate muscles. The presence of dense SEC or thrombus was verified by two independent observers.

### Speckle-Tracking Echocardiography

All strain analysis was performed with vendor-dependent software (EchoPAC PC version 203, GE Vingmed Ultrasound AS, Horten Norway). The LA and LAA endocardial borders were manually traced in apical four-chamber and two-chamber views (LA) and mid-esophageal views obtained at 0°, 45°, 90°, and 135°(LAA), respectively. Regions of interest were manually adjusted to fit the wall thickness. All tracking was reviewed to ensure it truly represented LA/LAA wall motion, and poorly tracked segments would be rejected. The strain curves of the global and regional LA /LAA wall were generated, and global peak positive longitudinal strain (GLS) was measured and averaged from two apical views for LA and four mid-esophageal views for LAA. LAA and LA MD was defined as the standard deviation (SD) of the time to peak positive strain of each segment and expressed as a percentage of the R-R’ interval. Higher values of MD indicate a greater degree of mechanical dyssynchrony. The reference frame of zero strain was set at left ventricular (LV) end-diastole (R-R gating) (29). To resolve the problem of beat-to-beat variation in STE measurements we used the index-beat method (22, 30). A cardiac cycle was selected for analysis where the preceding and pre-preceding R-R interval are of similar duration. All echocardiographic analysis was performed by one investigator experienced with strain imaging and blinded to the patients’ information. Among the 11,832 LAA segments and 5,916 LA segments analyzed, STE analysis was feasible in 16,926 (95.4%) segments.

### Statistical Analysis

IBM SPSS package 25.0 (SPSS, Inc., Chicago, IL, United States) and MedCalc version 12.5.0.0 (MedCalc Software, Mariakerke, Belgium). was used to perform the statistical analyses. Statistical significance was defined as *P* < 0.05. Continuous data were presented as mean ± standard deviation for normally distributed variables and median (interquartile range) for non-Gaussian variables. Categorical variables were summarized as number and percentages. Comparisons of the variables were performed by using independent Student’s *t*-test, the Mann-Whitney *U* test, Chi-square test or Fisher’s exact test where appropriate. Multivariate binary logistic regression analysis was performed to determine the independent markers using variables with *p* < 0.05 in the univariate analysis. Receiver operating characteristic (ROC) curves for different variables to test their abilities to discriminate patients with and without LAA dense SEC/thrombus, and the optimal cutoff value was determined as the value closest to the corner of the ROC curve. The incremental value of LAA MD and other mechanical parameters was tested by comparing global χ^2^ values in a series of models, areas under the ROC curve (AUCs) and net reclassification improvement.

Inter- and intra-observer variability for LA/LAA GLS and LA/LAA MD were studied in a random sample of 25 patients. Measurements were repeated >4 weeks apart by the same observer and by another experienced reader. The mean absolute differences between repeated measurements were calculated and assessed using Bland-Altman plots.

## Results

### Demographic and Clinical Characteristics

Out of 656 patients with AF, we excluded 58 patients either with congenital heart disease (*n* = 10), history of cardiac surgery and/or cardiac device implantation (*n* = 12), cardiac mass (*n* = 3), cardiomyopathies (*n* = 16), and inadequate image quality to perform strain analysis (*n* = 17). We also excluded patients who were in sinus rhythm at the time of echocardiography (*n* = 105). A total of 493 patients were included in the final analysis [median age, 65 (58.5, 71.0) years; 33.1% women, 47.5% persistent AF]. A total of 70 (14.2%) patients had dense SEC in LAA, while 38 (54.3%) had thrombus in LAA. The remaining patients were designated as the control group (*n* = 423, 85.8%). Table 1 summarizes clinical characteristics of the study population. Patients with dense SEC/thrombus were older, with higher CHA2DS2-VASc scores, higher incidence of persistent AF, previous TIA or stroke and heart failure. In addition, patients in the SEC/thrombus group had significantly higher plasma homocysteine levels. A total of 236 (47.9%) patients were on anticoagulation prior to ablation, and the usage of anticoagulants didn’t differ between two groups (*P* = 0.367). According to the standard of peri-procedural care for catheter ablation of AF, all patients were on anticoagulation at the time of ablation procedure. TEE was normally performed several hours before the ablation and anticoagulants were not discontinued. In patients not receiving anticoagulation (*n* = 257), 125 had a CHADS-VASc score ≥2. The underuse of anticoagulants in these patients were due to poor compliance, high bleeding risk, or other contraindications.

**TABLE 1:** Baseline clinical characteristics.

Clinical characteristic	All patients (*n* = 493)	Dense SEC or thrombus (*n* = 70)	Controls (*n* = 423)	*P*-value
Age, y	65 (58.5–71.0)	69.5 (63.8–74.3)	64 (58–71)	<0.01
Female	163 (33.1)	49 (70)	281 (66.4)	0.56
Body mass index, kg/m^2^	24.5 (22.3–26.7)	24.4 (22.3–26.3)	24.5 (22.4–26.7)	0.55
Body surface area, m^2^	1.7 (1.6–1.9)	1.7 (1.6–1.8)	1.7 (1.6–1.9)	0.12
CHA2DS2-VASc score	2 (1–3)	3 (1.8–4)	2 (1–3)	<0.01
Persistent AF	234 (47.5)	56 (80.0)	178 (42.1)	<0.01
Prior stroke/TIA	72 (14.6)	19 (27.1)	53 (12.5)	0.01
Anticoagulation	236 (47.9)	37 (52.9)	199 (47.1)	0.37
Warfarin	82 (16.6)	17 (24.3)	65 (15.4)	
Rivaroxaban	125 (25.4)	13 (18.6)	112 (26.5)	
Dabigatran	29 (5.9)	7 (10)	22 (5.2)	
Heart failure	37 (7.5)	12 (17.1)	25 (5.9)	0.001
LV ejection fraction < 50%	24 (4.9)	11 (15.7)	13 (3.1)	
Old myocardial infarction	5 (1)	1 (1.4)	4 (0.9)	0.71
Coronary artery disease	77 (15.6)	16 (22.9)	61 (14.4)	0.07
Hypertension	291 (59.0)	48 (68.6)	243 (57.4)	0.08
Diabetes	82 (16.6)	11 (15.7)	71 (16.8)	0.82
Dyslipidemia	169 (34.3)	19 (27.1)	150 (35.5)	0.17
Homocysteine, μmol/L	11.8 (9.7–14.6)	13.3 (10.7–17.0)	11.7 (9.4–14.1)	<0.01

*Data are expressed as median (interquartile range), or number (percentage).*

*AF, atrial fibrillation; LV, left ventricle; SEC, spontaneous echo contrast; TIA, transient ischemic attack.*

### Echocardiographic Parameters

**FIGURE 1:**
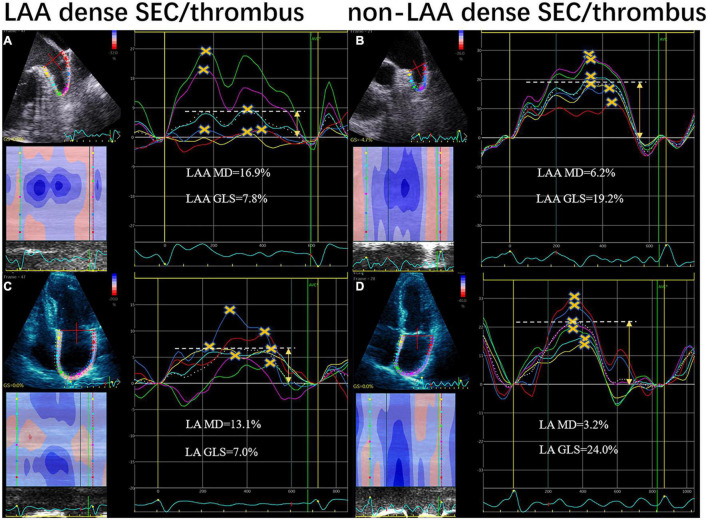
LAA and LA strain curves from speckle-tracking in patients with LAA dense spontaneous echo contrast and thrombus **(A,C)** and controls **(B,D)**. Yellow crosses indicate the positive peaks of each curve. Yellow arrows indicate the global peak longitudinal strain (GLS). Mechanical dispersion (MD) was defined as the SD of time to peak and expressed as a percentage of the R-R interval. LA, left atrium; LAA, left atrial appendage.

Table 2 shows a comparison of echocardiographic parameters between the patients with and without dense SEC or thrombus. Although LV volumes were comparable between two groups, the patients with dense SEC or thrombus had significantly lower LV ejection fraction (EF). Patients in this group also had increased LA /LAA volumes index (LAVI, LAAVI), decreased LA/LAA function (presented as emptying fraction and GLS), and reduced LAA flow velocities. Furthermore, LAA MD were more pronounced in SEC/thrombus group than in the controls [median 9.4 (6.2–12.1)% vs. 14.2 (11.6–16.8)%, respectively; *P* < 0.01]. Figure 1 shows representative LA and LAA strain curves in patients with and without dense SEC or thrombus.

**TABLE 2:** Echocardiographic parameters by groups.

Parameters	All patients (*n* = 493)	Dense SEC or thrombus (*n* = 70)	Controls (*n* = 423)	*P*-value
**LV parameters**				
LV end-diastolic volume index, mL/m^2^	65.6 ± 14.2	68.5 ± 16.9	65.1 ± 13.7	0.11
LV end-systolic volume index, mL/m^2^	22.3 (17.7–27.7)	24.0 (18.1–33.8)	22.0 (17.8–27.3)	0.06
LV mass index, g/m^2^	97.5 (84.2–115.8)	103.7 (91.8–123.2)	96.0 (83.4–114.1)	0.03
LV ejection fraction, %	64.9 (59.4–70.3)	61.9 (56.3–69.0)	65.3 (59.9–70.3)	0.01
**LA parameters**				
LAVI_max_, mL/m^2^	42.0 (32.3–53.8)	58.5 (48.6–69.0)	40.5 (31.2–49.5)	<0.01
LAVI_min_, mL/m^2^	26.1 (16.6–40.0)	47.0 (35.7–58.2)	24.4 (16.0–36.1)	<0.01
LA emptying fraction, %	35.6 (22.5–50.0)	21.8 (15.0–28.5)	38.2 (24.6–51.8)	<0.01
LA GLS, %	14.6 (9.5–25.5)	9.0 (7.1–11.0)	17.5 (10.9–26.8)	<0.01
LA MD, %	8.0 (5.5–10.4)	10.2 (8.1–13.5)	7.7 (5.2–9.9)	<0.01
**LAA parameters**				
LAAVI_max_, mL/m^2^	3.5 (2.5–4.7)	5.0 (3.7–7.3)	3.3 (2.4–4.2)	<0.01
LAAVI_min_, mL/m^2^	1.4 (0.6–2.5)	3.5 (2.1–4.3)	1.2 (0.6–2.1)	<0.01
LAA emptying fraction, %	55.6 (40.0–71.4)	37.5 (22.6–50.0)	60 (45.5–75.0)	<0.01
LAA emptying velocity, cm/s	46.0 (33.0–63.0)	28.0 (24.0–36.0)	49.0 (36.3–66.0)	<0.01
LAA filling velocity, cm/s	51.0 (38.0–66.0)	32.5 (24.0–44.8)	54.0 (42.0–68.0)	<0.01
LAA GLS, %	11.8 (8.7–16.1)	7.8 (6.0–9.3)	12.4 (9.4–16.8)	<0.01
LAA MD, %	9.9 (6.7–12.7)	14.2 (11.6–16.8)	9.4 (6.2–12.1)	<0.01

*Data are expressed as mean ± SD or median (interquartile range).*

*GLS, global longitudinal strain; LA, left atrium; LAA, left atrial appendage; LAAVI, LAA volume index; LAVI, LA volume index; LV, left ventricle; MD, mechanical dispersion; SEC, spontaneous echo contrast.*

### Determinants of Left Atrial Appendage Dense Spontaneous Echo Contrast or Thrombus

In the univariate logistic regression analysis, LAA dense SEC or thrombus was significantly associated with various clinical and echocardiographic parameters (Table 3). LAA MD remained to be independent in four different multivariate models (OR 1.23–1.24, *p* < 0.01) (Table 4). We also confirmed the independent association of homocysteine (OR 1.05–1.07, *p* < 0.05), LA GLS (OR 0.87–0.89, *p* < 0.01), LAAFV (OR 0.94–0.95, *p* < 0.01), and LAA GLS (OR 0.82–0.89, *P* < 0.05) with dense SEC or thrombus in at least two models. However, LA MD was not an independent contributor in any multivariate models.

**TABLE 3:** Univariable analyses of associations with LAA dense SEC or thrombus.

Variables	OR (95% CI)	*P*-value
**Clinical parameters**		
Age, y	1.06 (1.03–1.09)	<0.01
Female	0.85 (0.49–1.47)	0.56
Body mass index, kg/m^2^	0.97 (0.89–1.05)	0.39
CHA2DS2-VASc score	1.36 (1.16–1.60)	<0.01
Persistent AF	5.51 (2.97–10.2)	<0.01
Prior stroke/TIA	2.60 (1.43–4.74)	0.002
Anticoagulation	1.26 (0.76–2.10)	0.37
Heart failure	3.29 (1.57–6.91)	0.002
Homocysteine	1.07 (1.03–1.11)	<0.01
**Echocardiographic parameters**		
LV end-diastolic volume index, mL/m^2^	1.02 (0.99–1.04)	0.06
LV end-systolic volume index, mL/m^2^	1.05 (1.02–1.07)	<0.01
LV mass index, g/m^2^	1.01 (1.00–1.02)	0.04
LV ejection fraction, %	0.95 (0.93–0.98)	0.001
LAVI_max_, mL/m^2^	1.07 (1.05–1.09)	<0.01
LAVI_min_, mL/m^2^	1.08 (1.06–1.10)	<0.01
LA emptying fraction, %	0.94 (0.92–0.96)	<0.01
LA GLS, %	0.83 (0.78–0.88)	<0.01
LA MD, %	1.26 (1.17–1.35)	<0.01
LAAVI_max_, mL/m^2^	1.57 (1.37–1.79)	<0.01
LAAVI_min_, mL/m^2^	2.43 (1.97–2.99)	<0.01
LAA emptying fraction, %	0.94 (0.93–0.96)	<0.01
LAA emptying velocity, cm/s	0.91 (0.88–0.93)	<0.01
LAA filling velocity, cm/s	0.92 (0.90–0.94)	<0.01
LAA GLS, %	0.68 (0.62–0.76)	<0.01
LAA MD, %	1.29 (1.21–1.37)	<0.01

*AF, atrial fibrillation; GLS, global longitudinal strain; LA, left atrium; LAA, left atrial appendage; LAAVI, LAA volume index; LAVI, LA volume index; LV, left ventricle; MD, mechanical dispersion; CI, Confidence intervals; OR, odds ratio; SEC, spontaneous echo contrast; TIA, transient ischemic attack.*

**TABLE 4:** Multivariate analyses of associations with LAA dense SEC or thrombus.

Variable	Model 1		Model 2		Model 3		Model 4	
	OR (95% CI)	*P*-value	OR (95% CI)	*P*-value	OR (95% CI)	*P*-value	OR (95% CI)	*P*-value
**Clinical parameters**								
Age	1.02 (0.98, 1.06)	0.45	1.01 (0.97–1.06)	0.57	1.02 (0.98–1.06)	0.26		
CHA2DS2-VASc score	0.99 (0.76–1.29)	0.94	0.99 (0.75–1.31)	0.97	1.02 (0.79–1.32)	0.86	1.01 (0.79–1.28)	0.96
Persistent AF	1.17 (0.50–2.72)	0.72	1.04 (0.41–2.62)	0.94	1.25 (0.54–2.91)	0.60		
Homocysteine	1.05 (1.01–1.10)	0.02	1.07 (1.02–1.12)	0.009	1.06 (1.01–1.10)	0.02	1.07 (1.02–1.13)	0.006
**LV parameters**								
LV end-systolic volume index, mL/m^2^					1.00 (0.94–1.07)	0.93	1.01 (0.97–1.04)	0.71
LV mass index, g/m^2^					1.01 (0.99–1.02)	0.35		
LV ejection fraction, %					0.99 (0.95–1.03)	0.61	1.00 (0.93–1.07)	0.94
**LA parameters**								
LAVI_max_, mL/m^2^	1.02 (1.00–1.05)	0.05					1.01 (0.98–1.04)	0.45
LA emptying fraction, %	0.99 (0.96–1.02)	0.59						
LA GLS, %	0.89 (0.83–0.97)	0.005	0.93 (0.85–1.01)	0.08	0.87 (0.81–0.94)	<0.01	0.92 (0.85–1.00)	0.05
LA MD, %	1.05 (0.95–1.16)	0.37	1.10 (0.99–1.23)	0.09	1.07 (0.96–1.18)	0.23	1.11 (0.99–1.24)	0.07
**LAA parameters**								
LAAVI_max_, mL/m^2^			1.18 (0.98–1.43)	0.08				
LAA emptying fraction, %			0.99 (0.97–1.02)	0.50				
LAA emptying velocity, cm/s			0.99 (0.95–1.04)	0.77				
LAA filling velocity, cm/s			0.95 (0.92–0.97)	<0.01			0.94 (0.92–0.97)	<0.01
LAA GLS, %	0.84 (0.75–0.94)	0.002	0.90 (0.80–1.00)	0.06	0.82 (0.74–0.91)	<0.01	0.89 (0.79–0.99)	0.04
LAA MD, %	1.24 (1.14–1.35)	<0.01	1.23 (1.13–1.34)	<0.01	1.24 (1.14–1.35)	<0.01	1.24 (1.14–1.35)	<0.01

*AF, atrial fibrillation; GLS, global longitudinal strain; LA, left atrium; LAA, left atrial appendage; LAAVI, LAA volume index; LAVI, LA volume index; LV, left ventricle; MD, mechanical dispersion; CI, Confidence intervals; OR, odds ratio; SEC, spontaneous echo contrast.*

*Model 1 adjusted with clinical and LA echocardiographic parameters.*

*Model 2 adjusted with clinical and LAA echocardiographic parameters.*

*Model 3 adjusted with clinical and LV echocardiographic parameters.*

*Model 4 adjusted with multi mixed parameters.*

ROC curve analysis results are listed in Supplementary Table 1. The AUCs for LAA parameters were higher than clinical, LV and LA variables. Importantly, the AUCs for LAA MD (0.82), LAA GLS (0.84), and LA GLS (0.80) were comparable but higher than that of LA MD (0.74, all *p* < 0.05). The optimal cutoff value for LAA MD to identify LAA dense SEC or thrombus was >11.2%, with a sensitivity of 80.0% and specificity of 67.16%. We also calculated cutoff values for LAA GLS, LA GLS, and LA MD.

### Incremental Value of Left Atrial Appendage Mechanical Dispersion for Identifying Dense Spontaneous Echo Contrast or Thrombus and Comparison With Other Left Atrial/Left Atrial Appendage Mechanics

**FIGURE 2:**
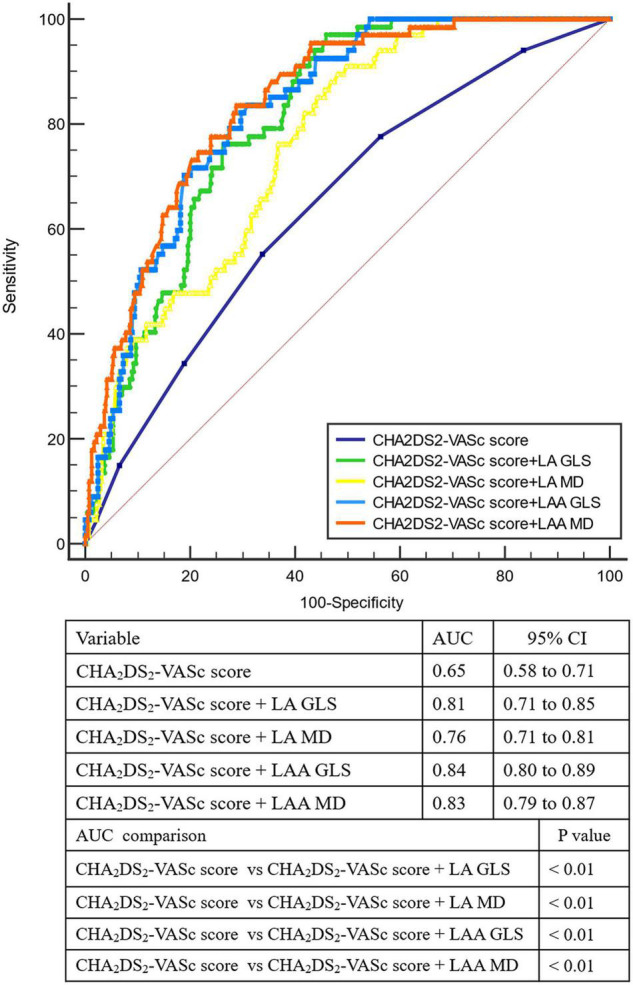
Receiver-operating characteristic curve analysis for identifying LAA dense spontaneous echo contrast or thrombus. AUC, area under the curve; GLS, global longitudinal strain; LA, left atrium; LAA, left atrial appendage; MD, mechanical dispersion.

**FIGURE 3:**
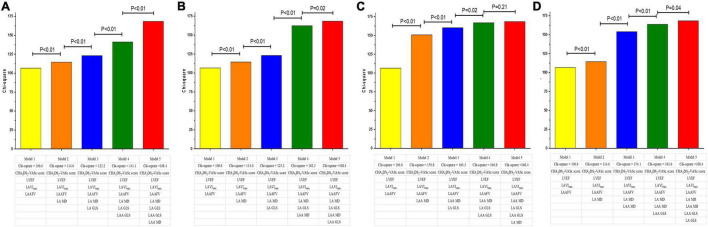
Incremental values of LAA and LA mechanics for risk stratification of LAA dense spontaneous echo contrast or thrombus. The initial model was based on CHA2DS2-VASc score, LVEF, LAVI_max_ and LAA FV. LA MD, LAGLS, LAA GLS, and LAA MD was sequentially added to the initial model in different order and the global χ^2^ value was compared. The parameter added in the last step was LAA MD **(A)**, LAA GLS **(B)**, LA MD **(C)**, and LA GLS **(D)**. The addition of LA MD provided no incremental value over other LA/LAA mechanical parameters. GLS, global longitudinal strain; LA, left atrium; LAA, left atrial appendage; LAAFV, LAA filling velocity; LAVI, LA volume index; LVEF, left ventricular ejection fraction; MD, mechanical dispersion.

The addition of LA/LAA mechanics (LA GLS, LAA GLS, LA MD, LAA MD) to CHA2DS2-VASc score significantly improved AUCs in ROC curve analyses. Moreover, the AUC of CHA2DS2-VASc score plus LAA MD was significantly higher than adding LA MD (0.83 vs. 0.76, *P* = 0.04), but comparable to adding LAA GLS or LA GLS (*P* = 0.61 and 0.48, respectively, Figure 2). We also assessed the incremental value of LAA MD, LAA GLS, LA GLS or LA MD over one another by comparing the global χ^2^ value in modeling steps. The initial model based on CHA2DS2-VASc score, LVEF, LAVI_max_, and LAAFV (χ^2^ = 106.6) was significantly improved by adding LA MD (χ^2^ = 114.6, *P* < 0.01) and further improved by adding LA GLS (χ^2^ = 123.2, *P* < 0.01), LAA GLS (χ^2^ = 141.1, *P* < 0.01) and finally LAA MD (χ^2^ = 168.4, *P* < 0.01). Similarly, the same initial model was significantly improved by adding LA GLS (*P* = 0.04) and LAA GLS (*P* = 0.02) in the last step of sequential models (Figure 3). However, the addition of LA MD provided no incremental value (*P* = 0.21) over other LA/LAA mechanical parameters.

**FIGURE 4:**
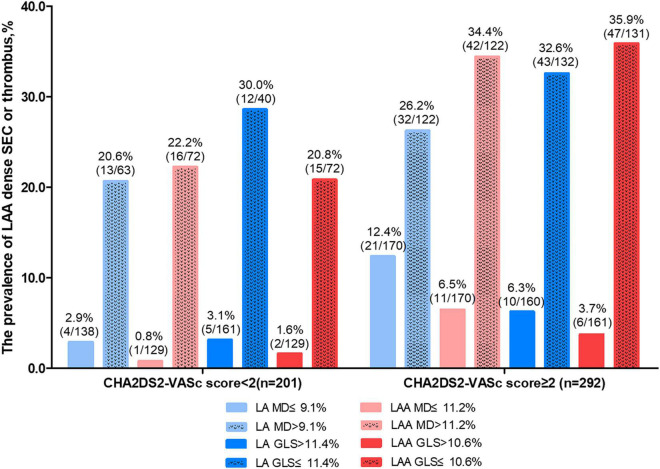
The prevalence of LAA dense spontaneous echo contrast or thrombus according to LA/LAA mechanics and CHA2DS2-VASc score. GLS, global longitudinal strain; LA, left atrium; LAA, left atrial appendage; MD, mechanical dispersion.

Moreover, we summarized the prevalence of LAA dense SEC or thrombus according to different LA /LAA mechanics and CHA2DS2-VASc score (Figure 4). LAA MD significantly increased the risk of LAA blood stasis in patients with CHA2DS2-VASc score <2 (OR 36.3, 95% CI 4.7–280.4, *P* = 0.001) (Supplementary Table 2). Adding LAA MD, LAA GLS, and LAGLS to CHA2DS2-VASc score led to significant net reclassification improvement (0.30, 0.27 and 0.26, all *P* < 0.01) whereas adding LA MD did not lead to significant improvement (0.17, *P* = 0.06) (Supplementary Tables 3–6).

### Reproducibility

Bland-Altman analysis for inter-and intra-observer variability was shown in Supplementary Figure 1. For inter-observer reproducibility, the mean differences in LA GLS, LAA GLS, LA MD, and LAA MD were 1.1 ± 2.7%, 0.7 ± 3.0%, 2.1 ± 2.3% and 3.0 ± 3.5%, respectively. The mean differences between the repeated measures of the same observer in LA GLS, LAA GLS, LA MD and LAA MD were −0.7 ± 2.8%, −0.6 ± 2.5%, 0.8 ± 3.1%, and 2.4 ± 3.8%, respectively.

## Discussion

In this cross-sectional study, we found that LAA mechanical dispersion assessed by speckle-tracking echocardiography was an independent determinant of LAA dense SEC or thrombus in patients with AF, incremental to clinical and echocardiographic parameters and other strain measurements. This study also identified similarly significant reclassification improvement by adding LAA MD, LAA GLS, and LA GLS to CHA2DS2-VASc score. Moreover, LAA MD was superior to LA MD in identifying LAA dense SEC or thrombus.

### Left Atrial Appendage Mechanical Dispersion as a Mechanism of Thrombogenesis

As the most common site for thrombus formation (7), LAA should be given meticulous attention in the setting of AF. In line with previous results (20, 21, 31), our findings confirmed that LAA EF, LAA flow velocities and LAA GLS are significant contributors to LAA stasis, with the latter two remaining independent in at least two multivariate models. We also found AF patients with dense SEC or thrombus in LAA had higher LAA MD than those without. The LAA MD cutoff of >11.2% help clinicians to sensitively identify patients at higher risk for LAA dense SEC or thrombus, especially in those with CHA2DS2-VASc score <2. To the best of our knowledge, this is the first study demonstrating that LAA MD is an independent determinant of LAA stasis in AF patients using speckle-tracking echocardiography. What’s more, LAA MD provided incremental values over clinical, conventional TTE and TEE parameters.

The mechanistic link as to how abnormal LAA MD causes thrombosis remains unclear. Previous studies found an association among disturbances in the LAA conduction, LAA fibrosis and LAA thrombus (9, 11, 32). We speculate that LAA fibrotic changes increase LAA discoordination during filling phase, which consequently slow down the regional blood flow and lead to thrombogenesis. Reflecting this change, LAA MD might contribute to LAA blood stasis.

### Comparison of Predictive Values Among Left Atrial/Left Atrial Appendage Mechanics

Although the correlation of LA MD with LAA thrombus or sludge has been demonstrated previously (22, 23), our study revealed that LAA MD, rather than LA MD had an independent association with LAA dense SEC or thrombus. LA MD did not maintain its significance when adjusted for LAVI_max_ and LA GLS. As a sensitive marker of LA dysfunction and asynchrony, impairment of LA MD precedes morphological changes (33). In the present study, the majority of patients had LA enlargement (70.9%), indicating that most of them had experienced significant LA remodeling, hence, predictive value of LA MD became less pronounced compared with LA volume or LA GLS. In addition, mechanical discordance between LA and LAA existed in 25% of AF patients (34), suggesting that LA dispersion may not represent LAA wall motion discoordination and a dedicated analysis of LAA dyssynchrony would provide extra information. These findings indicated that LAA myocardial function should be assessed even in the presence of LA enlargement and dysfunction.

### Clinical Implications

The findings in this study suggest that LAA MD could potentially reduce underuse of anticoagulants by improving decision making for anticoagulation in patients at a high risk of LAA thrombus despite a low CHA2DS2-VASc score. LAA remodeling is partially reversible (35), and therapies aiming at LAA MD might potentially benefit patients by improving LAA mechanics and future studies are needed to validate this hypothesis. Although LAA MD is superior to LA MD in predicting LAA blood stasis, its assessment is more time-consuming and less validated than LA strain measurements. Given the comparable predictive value of LA GLS, it may be an alternative to LAA mechanics in patients who could not tolerate TEE or TEE images were inadequate for strain analysis.

### Study Limitations

There are several limitations and technical considerations in the present study. First of all, this is a cross-sectional, observational study consisting of patients referring for catheter ablation of AF. Therefore, selection bias should be taken into account. Second, given the complex and variable morphology of LAA, although we examined the LAA from four different views, it is still difficult to visualize its entirety and small thrombi within a side lobe might be overlooked. Third, vendor specificity of STE and lack of specific strain packages should be considered. Although we analyzed LA and LAA strain using software for evaluating the LV, the 2D strain package allowed manual adjustment of a region of interest to fit the thickness of LA and LAA wall. Fourth, the cutoff value of LAA MD was derived from ROC analysis and reported in the same derivation cohort, rather than in an independent group of patients. This cutoff should be externally verified by further prospective multicenter studies.

## Conclusion

Left atrial appendage mechanical dispersion assessed by speckle-tracking echocardiography was an independent determinant of LAA dense SEC or thrombus in AF patients, incremental to clinical risk factors and conventional echocardiographic parameters, and superior to LA mechanical dispersion.

## Data Availability Statement

The datasets used and/or analyzed during the current study are available from the corresponding author upon reasonable request.

## Ethics Statement

The studies involving human participants were reviewed and approved by the Sir Run Run Shaw Hospital, Zhejiang University School of Medicine, Hangzhou, China; First Affiliated Hospital, Zhejiang University School of Medicine, Hangzhou, China. The patients/participants provided their written informed consent to participate in this study.

## Author Contributions

CJ and ZZ: conceptualization and methodology. YM and HZ: data curation and writing – original draft preparation. YY and CY: investigation, visualization, and formal analysis. MM and BZ: visualization and writing – review and editing. YW: resources and validation. RJ: resources and funding acquisition. All authors contributed to the article and approved the submitted version.

## Conflict of Interest

The authors declare that the research was conducted in the absence of any commercial or financial relationships that could be construed as a potential conflict of interest.

## Publisher’s Note

All claims expressed in this article are solely those of the authors and do not necessarily represent those of their affiliated organizations, or those of the publisher, the editors and the reviewers. Any product that may be evaluated in this article, or claim that may be made by its manufacturer, is not guaranteed or endorsed by the publisher.
