# Supplementary material for: Left Atrial Appendage Mechanical Dispersion Assessed by Speckle-Tracking Echocardiography as a Determinant of Left Atrial Appendage Blood Stasis in Patients With Atrial Fibrillation
Source: Front Cardiovasc Med. 2022 Jun 6;9:905293. doi: 10.3389/fcvm.2022.905293 (PMC9207320; doi:10.3389/fcvm.2022.905293)
Supplement: Supplementary file 1 [file Data_Sheet_1.docx]

Supplementary Material

Table S1 AUC for ROC analysis of clinical and echocardiographic variables.

| Variables | All patients (N=493, dense SEC or thrombus =70) | | |
| --- | --- | --- | --- |
|  | AUC | 95%CI | P value |
| **Clinical parameters** |  |  |  |
| CHA_2_DS_2_-VASc score | 0.65 | 0.58-0.71 | <0.01 |
| Homocysteine | 0.64 | 0.58-0.71 | <0.01 |
| **LV parameters** |  |  |  |
| LVESV index, mL/m2 | 0.57 | 0.49-0.65 | 0.07 |
| LV mass index, g/m2 | 0.58 | 0.51-0.65 | 0.03 |
| LVEF, % | 0.59 | 0.52-0.67 | 0.02 |
| **LA parameters** |  |  |  |
| LAVI_max_, mL/m2 | 0.80 | 0.75-0.85 | <0.01 |
| LAEF, % | 0.77 | 0.72-0.82 | <0.01 |
| LA GLS, % | 0.80 | 0.76-0.85 | <0.01 |
| LA MD, % | 0.74 | 0.69-0.80 | <0.01 |
| **LAA parameters** |  |  |  |
| LAAVI_max_, mL/m2 | 0.75 | 0.69-0.81 | <0.01 |
| LAA EF, % | 0.81 | 0.77-0.86 | <0.01 |
| LAAEV, cm/s | 0.84 | 0.79-0.89 | <0.01 |
| LAAFV, cm/s | 0.81 | 0.75-0.86 | <0.01 |
| LAA GLS, % | 0.84 | 0.80-0.88 | <0.01 |
| LAA MD, % | 0.82 | 0.78-0.87 | <0.01 |

AUC, area under the curve; CI, Confidence intervals; GLS, global longitudinal strain; LA, left atrium; LAA, left atrial appendage; LAAEF, LAA emptying fraction; LAAEV, LAA emptying velocity; LAAVI, LAA volume index; LAEF, LA emptying fraction; LAVI, LA volume index; LV, left ventricle; LVEF, LV ejection fraction; LVESV, LV end-systolic volume; MD, mechanical dispersion; ROC, Receiver operating characteristic; SEC, spontaneous echo contrast.

Table S2 LAA/LA mechanics and risk of LAA dense SEC or thrombus in different CHA_2_DS_2_-VASc score

| CHA2DS2-VASc score<2(n=201) | OR | 95% CI | P-value |
| --- | --- | --- | --- |
| LA GLS, % | 12.3 | 4.3-48.7 | <0.01 |
| LA MD, % | 8.7 | 2.7-28.0 | <0.01 |
| LAA GLS, % | 16.7 | 3.8-76.9 | <0.01 |
| LAA MD, % | 36.3 | 4.7-280.4 | 0.001 |
| CHA2DS2-VASc score≥2(n=292) | OR | 95% CI | P-value |
| LA GLS, % | 7.3 | 3.6-15.9 | <0.01 |
| LA MD, % | 2.8 | 1.5-5.0 | 0.002 |
| LAA GLS, % | 14.5 | 5.8-34.6 | <0.01 |
| LAA MD, % | 7.6 | 3.3-12.9 | <0.01 |

GLS, global longitudinal strain; LA, left atrium; LAA, left atrial appendage; MD, mechanical dispersion; SEC, spontaneous echo contrast.

Table S3 Net reclassification table: The addition of LAA MD to CHA_2_DS_2_-VASc score

| Variables | CHA2DS2-VASc score  +LAA MD | | | Reclassified | |  |
| --- | --- | --- | --- | --- | --- | --- |
| No dense SEC or thrombus (n = 423) | Low risk | High risk |  | Increased risk* | Decreased risk* | Net correctly reclassified (%) |
| CHA2DS2-VASc score |  |  |  |  |  |  |
| Low risk | 133 | 51 |  | 51 | 128 | 18.2 |
| High risk | 128 | 111 |  |  |  |  |
| Dense SEC or thrombus (n = 70) |  |  |  |  |  |  |
| CHA2DS2-VASc score |  |  |  |  |  |  |
| Low risk | 1 | 16 |  | 16 | 8 | 11.4 |
| High risk | 8 | 45 |  |  |  |  |
| Net reclassification improvement = 0.30(P<0.01) | | | | | | |

The risk for dense SEC or thrombus was stratified into low (0% to < 10%) and high risk (≥10%). The net reclassification improvement is the sum of correctly reclassified individuals with and without dense SEC or thrombus. *The number of individuals who were reclassified upward and downward, respectively.

GLS, global longitudinal strain; LA, left atrium; LAA, left atrial appendage; MD, mechanical dispersion; SEC, spontaneous echo contrast.

Table S4 Net reclassification table: The addition of LAA GLS to CHA2DS2-VASc score

| Variables | CHA2DS2-VASc score  +LAA GLS | | | Reclassified | |  |
| --- | --- | --- | --- | --- | --- | --- |
| No dense SEC or thrombus (n = 423) | Low risk | High risk |  | Increased risk* | Decreased risk* | Net correctly reclassified (%) |
| CHA2DS2-VASc score |  |  |  |  |  |  |
| Low risk | 135 | 49 |  | 49 | 127 | 18.4 |
| High risk | 127 | 112 |  |  |  |  |
| Dense SEC or thrombus (n = 70) |  |  |  |  |  |  |
| CHA2DS2-VASc score |  |  |  |  |  |  |
| Low risk | 6 | 11 |  | 11 | 5 | 8.6 |
| High risk | 5 | 48 |  |  |  |  |
| Net reclassification improvement = 0.27 (P<0.01) | | | | | | |

The risk for dense SEC or thrombus was stratified into low (0% to < 10%) and high risk (≥10%). The net reclassification improvement is the sum of correctly reclassified individuals with and without dense SEC or thrombus. *The number of individuals who were reclassified upward and downward, respectively.

GLS, global longitudinal strain; LA, left atrium; LAA, left atrial appendage; MD, mechanical dispersion; SEC, spontaneous echo contrast.

Table S5 Net reclassification table: The addition of LA MD to CHA2DS2-VASc score

| Variables | CHA2DS2-VASc score  +LA MD | | | Reclassified | |  |
| --- | --- | --- | --- | --- | --- | --- |
| No dense SEC or thrombus (n = 423) | Low risk | High risk |  | Increased risk* | Decreased risk* | Net correctly reclassified (%) |
| CHA2DS2-VASc score |  |  |  |  |  |  |
| Low risk | 142 | 42 |  | 42 | 90 | 11.3 |
| High risk | 90 | 149 |  |  |  |  |
| Dense SEC or thrombus (n = 70) |  |  |  |  |  |  |
| CHA2DS2-VASc score |  |  |  |  |  |  |
| Low risk | 5 | 12 |  | 12 | 8 | 5.7 |
| High risk | 8 | 45 |  |  |  |  |
| Net reclassification improvement = 0.17(P=0.06) | | | | | | |

The risk for dense SEC or thrombus was stratified into low (0% to < 10%) and high risk (≥10%). The net reclassification improvement is the sum of correctly reclassified individuals with and without dense SEC or thrombus. *The number of individuals who were reclassified upward and downward, respectively.

GLS, global longitudinal strain; LA, left atrium; LAA, left atrial appendage; MD, mechanical dispersion; SEC, spontaneous echo contrast.

Table S6 Net reclassification table: The addition of LA GLS to CHA2DS2-VASc score

| Variables | CHA2DS2-VASc score  +LA GLS | | | Reclassified | |  |
| --- | --- | --- | --- | --- | --- | --- |
| No dense SEC or thrombus (n = 423) | Low risk | High risk |  | Increased risk* | Decreased risk* | Net correctly reclassified (%) |
| CHA2DS2-VASc score |  |  |  |  |  |  |
| Low risk | 132 | 52 |  | 52 | 115 | 14.9 |
| High risk | 115 | 124 |  |  |  |  |
| Dense SEC or thrombus (n = 70) |  |  |  |  |  |  |
| CHA2DS2-VASc score |  |  |  |  |  |  |
| Low risk | 4 | 13 |  | 13 | 5 | 11.4 |
| High risk | 5 | 48 |  |  |  |  |
| Net reclassification improvement = 0.26 (P<0.01) | | | | | | |

The risk for dense SEC or thrombus was stratified into low (0% to < 10%) and high risk (≥10%). The net reclassification improvement is the sum of correctly reclassified individuals with and without dense SEC or thrombus. *The number of individuals who were reclassified upward and downward, respectively.

GLS, global longitudinal strain; LA, left atrium; LAA, left atrial appendage; MD, mechanical dispersion; SEC, spontaneous echo contrast.

Figure legends

**Figure S1** Bland-Altman plots of intraobserver agreement for LAA MD(A), LAA GLS(B), LA MD(C) and LA GLS (D), and interobserver agreement for LAA MD(E), LAA GLS(F), LA MD(G) and LA GLS (H). GLS, global longitudinal strain; LA, left atrium; LAA, left atrial appendage; MD, mechanical dispersion.
