# Supplementary figures and images for: Left Atrial Appendage Mechanical Dispersion Assessed by Speckle-Tracking Echocardiography as a Determinant of Left Atrial Appendage Blood Stasis in Patients With Atrial Fibrillation
Source: Front Cardiovasc Med. 2022 Jun 6;9:905293. doi: 10.3389/fcvm.2022.905293 (PMC9207320; doi:10.3389/fcvm.2022.905293)

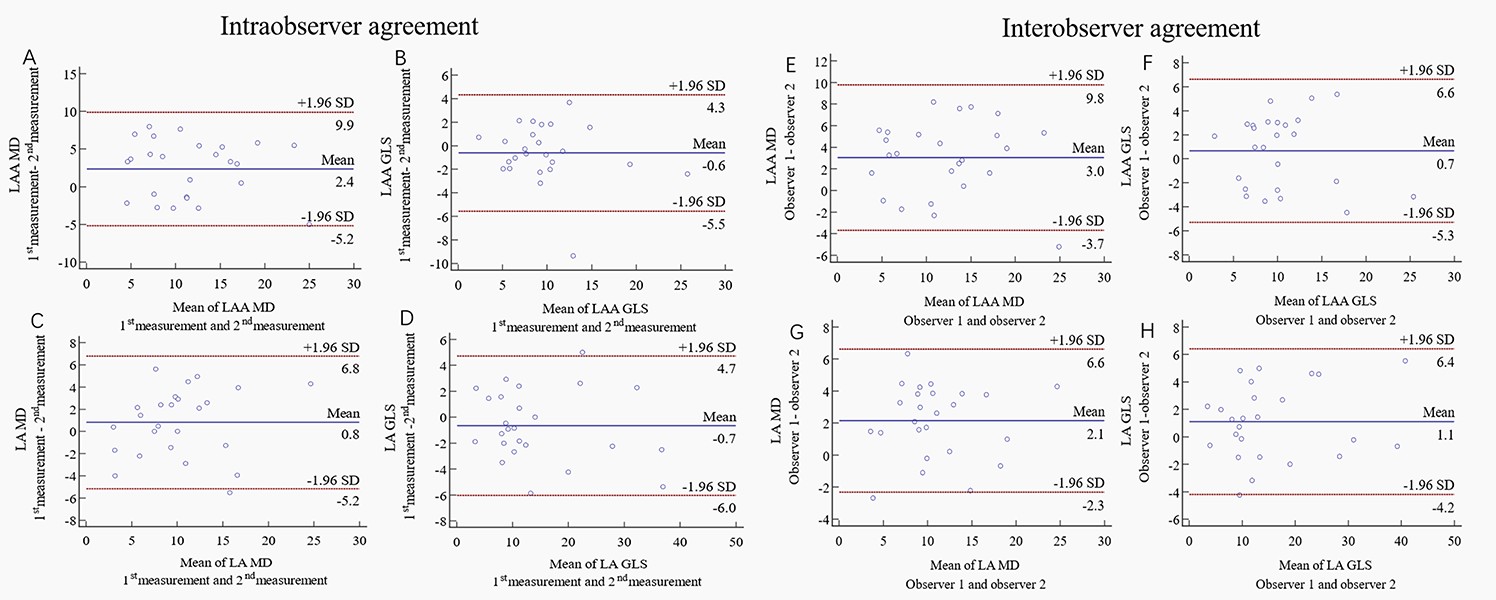

Supplement: Supplementary file 2 [file Image_1.TIF]
